# Inference by Exclusion in Goffin Cockatoos (*Cacatua goffini*)

**DOI:** 10.1371/journal.pone.0134894

**Published:** 2015-08-05

**Authors:** Mark O’Hara, Alice M. I. Auersperg, Thomas Bugnyar, Ludwig Huber

**Affiliations:** 1 Department of Cognitive Biology, University of Vienna, Vienna, Austria; 2 Messerli Research Institute, University of Veterinary Medicine Vienna, Medical University Vienna, University of Vienna, Vienna, Austria; 3 Behavioural Ecology Research Group, Department of Zoology, Oxford University, Oxford, United Kingdom; Centre national de la recherche scientifique, FRANCE

## Abstract

Inference by exclusion, the ability to base choices on the systematic exclusion of alternatives, has been studied in many nonhuman species over the past decade. However, the majority of methodologies employed so far are hard to integrate into a comparative framework as they rarely use controls for the effect of neophilia. Here, we present an improved approach that takes neophilia into account, using an abstract two-choice task on a touch screen, which is equally feasible for a large variety of species. To test this approach we chose Goffin cockatoos (*Cacatua goffini*), a highly explorative Indonesian parrot species, which have recently been reported to have sophisticated cognitive skills in the technical domain. Our results indicate that Goffin cockatoos are able to solve such abstract two-choice tasks employing inference by exclusion but also highlight the importance of other response strategies.

## Introduction

Animals frequently face situations in which only partial information about the problems at hand is immediately accessible. To cope with such situations individuals may employ different techniques involving various levels of cognition [1]. When faced with two or more alternatives, the simplest response strategy could be a random choice or, alternatively, generalisation from previously rewarding situations based on similarity, thereby using information from trial and error learning [2]. A cognitively more challenging strategy involves the analytical interpretation of the outcome of an unknown event using previous experience [3]. One way to reason in this manner is to infer by exclusion, i.e., choosing one option by logically excluding other alternatives [4].

Until recently it was believed that the ability to infer by exclusion might be associated with complex language learning and therefore a uniquely human trait [5,6]. However, in the past decade, extensive research on various different species has proved otherwise (primates [4,7–19], dogs (*Canis familiaris*) [7,20–22], dolphins (*Tursiops truncatus*) and sea lions (*Zalophus californianus*) [23–25], dwarf goats (*Capra aegagrus hircus*) and sheep (*Ovis orientalis aries*) [26], as well as corvids and parrots: [27–34]). In terms of ecological relevance, inference abilities have mainly been discussed in a foraging context (e.g. [15,26,30,31,33]). Nevertheless, other factors have also been considered as possible contributors to the emergence of this capacity, such as rejection of parasitic eggs [35], social complexity [16] or predator detection [27].

Studies on inference by exclusion have employed a wide range of different tasks in order to explore exclusion skills in various animal species. Perhaps the most common method, as originally devised by Call [8], required apes to locate hidden food items underneath two opaque cups. Test subjects were provided with partial information about these cups (either showing them the content of the cups, or shaking: both cups, only the baited cup, only the empty cup or neither cup). By giving individuals of the four great ape species—bonobos (*Pan paniscus*), chimpanzees (*Pan troglodytes*), gorillas (*Gorilla gorilla*) and orangutans (*Pongo pygmaeus*)—information (visual, by showing the inside of the cups, or auditory, by shaking the cups) about the content of both cups or only the baited cup the authors controlled for the motivation to retrieve the reward and choice based purely on manipulation of the cups. A further control condition, in which neither of the cups was manipulated, was devised to investigate whether individuals used other cues (e.g. olfaction). The crucial condition though was providing the test subjects only with information about the non-baited cup, which required individuals to choose by inferring the location of the hidden food reward without direct information. Follow-up experiment that eliminated alternative mechanisms to locate the food (such as learning to respond correctly, or simply associating cues with the reward), provided further support for Call's [8] previous findings.

Nawroth et al. [26] have extended the investigation of inference by exclusion skills also to non-primate mammals by providing goats and sheep with the same task in the visual domain. In a critical experiment the authors showed that two goats but no sheep chose the correct cup significantly above chance levels. The authors discuss this possible species difference with regard to the species-specific feeding ecology, with sheep being dietary grazers, whereas goats forage more selectively.

A task by Call and Carpenter [9] that was adapted to the visual domain by using straight and bent tubes, was implemented by Schloegl and colleagues [30] to investigate exclusion performance in ravens (*Corvus corax*) and kea (*Nestor notabilis*). Here, subjects were able to see a reward if it was located in a straight tube with the openings oriented towards them, but not if the reward was placed in a bent tube or a straight tube that was rotated so that the openings were visually not directly accessible. Thus the individuals were required to look into the tubes in order to find the reward. By counting the looks into relevant and irrelevant tubes the authors were able to determine whether the birds were applying exclusion skills. In total the raven seemed to show significantly more exclusion-based choices, while kea were also significantly more likely to choose the correct tube, but only after visually inspecting both tubes. In fact the kea even looked into straight tubes from both sides in approximately 35% of all trials, a response that is really not suggestive of reasoning by exclusion capacities in this species. Schloegl et al. [30,33,36] proposed that different selection pressures might have been responsible for these differences: while food storing and competitiveness were discussed as the driving forces behind inference by exclusion in ravens, the kea's explorative nature may have hindered this trait from emerging [30,33,36]. However it is also possible that their explorative and neophilic tendencies might have only overshadowed the kea's propensity to reason by exclusion and that the task therefore was not fully suited for a direct comparison between ravens and kea because it did not control for neophilia. Schloegl et al. [32] later discussed the possibility of domain-specific advantages for different species and highlighted the importance of procedural differences in a study showing that African Grey parrots (*Psittacus erithacus*) are able to perform reasoning by exclusion with acoustic cues.

Greenberg and Mettke-Hofmann [37] presented and tested an innovative theoretical model regarding exploration and neophobia, based on ecological factors. This two-factor model makes predictions when bird species will show i) more or less explorative tendencies and ii) neophilic or neophobic responses. The authors conclude that especially island species might exhibit the highest levels of exploration and neophilia as result of lower predation risk and exploitation of larger feeding niches. By that logic, island species such as the Goffin cockatoo should be a good model species to study the impact of neophilia and exploration on cognition and decision-making [38]. Neophilia, the attraction to objects or stimuli simply due to the fact that they are novel [39], has been discussed as a possible confounding factor in a variety of cognitive testing approaches in kea [40], another parrot species endemic to an island. However, implications of novelty seeking, with regards to cognitive testing in other species have so far, to our knowledge, been given little attention.

An abstract task using touch screens is a suitable approach to investigate species with different levels of neophobia and neophilia [41]. Aust et al. [7] devised an elaborate two-choice task on the touch screen in order to investigate inference by exclusion, while controlling for neophilic responses in humans, dogs and pigeons (*Columba livia*). They presented the individuals with a set of rewarded (S+) and unrewarded (S-) stimuli. Once the subjects reliably discriminated between the stimuli, they then introduced a novel stimulus (S') paired with the familiar S-, thus leaving two possible explanations for choosing the novel stimulus: either by excluding the former unrewarded S-, or choosing the novel stimulus due to a neophilic tendency. To be able to exclude the latter explanation the researchers controlled for neophilia by pairing the S' with yet another novel stimulus (S"). If subjects now shifted their responses to the S", then their responses would be guided by neophilic tendencies, whereas perseverative responses towards the S' would imply inference by exclusion. While most of the humans and half of the dogs in the study seemed to choose novel stimuli based on exclusion, only one pigeon chose the novel stimulus in the first test significantly above chance, but chose the second novel stimulus in the following test as well and thus was exhibiting neophilia. Despite the innovative rationale of this task, the procedure still had some serious shortcomings and limitations as discussed by Aust et al. [7]. Firstly and perhaps most importantly, test trials were not rewarded, which may have violated the expectations of individuals who initially performed according to the rationale of the task, but may have immediately abandoned this strategy following the absence of food rewards. Secondly, the experimental design of this task only allows mutually exclusively testing for either neophilic or exclusion strategies, but not both. If individuals fail in the second task, it may indicate that a correct response in the first test was guided by neophilic tendencies rather than exclusion skills. Finally, in contrast to the more traditional cup task of Call [8], which investigated this ability in a more ecologically valid setting, the study of Aust et al. [7] required the birds to first learn to associate certain stimuli with a reward. As this task involved training, and test stimuli were shown repeatedly, mere exposure might have influenced individuals' choices, whereas choices in a cup task may be more spontaneous. Still, an abstract task may be more suitable to investigate whether reasoning abilities can be applied in a more general way.

Here we present a modified procedure which partially adapts the basic idea of Aust and colleagues' [7] study, but at the same time controls for the first two of the above-mentioned shortcomings. We accomplished this by already introducing novelty trials during the training to habituate subjects to novel stimuli. However this training could result in a learned rule to avoid novel stimuli in general, despite the context in which the novel stimulus is presented. Violations of expectancies, promoted by not receiving a reward after selecting the logically "correct" stimulus might have accounted for weaker performance in later test sessions in the study of Aust et al. [7]. To overcome this effect, we decided to reward critical test trials differentially. To discourage the birds from forming stimulus-reward associations we presented novel stimuli in every session. Furthermore, we introduced a new condition to additionally exclude the possibility of one-trial learning as originally introduced by Guthrie [42] (see Procedure). Preferences for or avoidance of particular stimuli could also determine choices between stimuli. With this protocol we wanted to test whether subjects exhibit exclusion skills, but also to what extent alternative strategies would be employed. We chose the Goffin cockatoo (*Cacatua goffini*) as a pilot subject species for several reasons: They are not only highly inquisitive and explorative in captivity [43,44], but also have exceptional skills in tool manufacture and use [45,46], can solve stage six Piagetian object-permanence [43] and have already shown some functional inferences in a sequential means-end task [47].

There are many ecologically relevant domains in which inference by exclusion may be adaptive for Goffin cockatoos, be it in a foraging context, dealing with social challenges, or in the technical domain when making inferences about the functionality of an action, as has been shown in the study of Auersperg et al. [47]. So far only little is known about these birds in the wild [48], so we can only speculate about the underlying ultimate mechanisms promoting such skills in this species. However, the question remains whether such exclusion abilities can be applied in a domain-general manner, thus being transferred from the original context, whatever it may be, and employed in an abstract task. We believe that in order to address the evolution of inferential reasoning abilities in a fair and sensible comparative framework across different strategies and species in the future, an abstract touch screen setup will be of great use, provided that differences in behavioural and ecological predispositions are appropriately controlled for.

## Material and Methods

### Ethical statement

The Animal Ethics and Experimentation Board of the Faculty of Life Sciences at the University of Vienna approved the study (Reference number: 2015–001). All subjects that participated in reported experiments were housed in accordance with the Austrian Federal Act on the Protection of Animals (Animal Protection Act—TSchG, BGBl. I Nr.118/2004). Furthermore, the animals’ wings were not clipped and they rather than being inside a box during touch screen testing, were sitting on a perch inside the experimental room, free to fly off any time they chose to. If the birds were not motivated to participate they were released back into the group compartment. As the present study was strictly non-invasive and based purely on behavioural observations, all experiments are classified as non-animal experiments in accordance with the Austrian Animal Experiments Act (§ 2, Federal Law Gazette No. 501/1989).

### Test subjects

Twelve individuals of Goffin cockatoos participated in this study (see Table 1). The whole group, consisting of 15 birds, was group-housed in a large, heated (20°C) indoor aviary (45 m^2^, 3 m to 6 m high) with an adjacent outdoor aviary (150 m^2^, 3 m to 4.5 m high), to which they had access all day at temperatures above 17°C and at least one and a half hours per day if temperatures rose above -2°C. The aviaries were enriched with branches, hiding places, bathing opportunities and wooden parrot toys. Individual birds were visually separated from the rest of the group in an indoor testing room (7.5 m^2^, 3m high) adjacent to the group aviary. Five individuals were completely naive to the touch screen, seven had prior experience with the touch screen by participating in a two choice task investigating the effect of exploration and neophilia on discrimination learning (O'Hara et al., unpublished data).

**Table 1:** List of individuals participating.

Individual	Hatched	Sex	Touch screen experience
Doolittle	2011	♂	Yes
Figaro	2007	♂	Yes
Fini	2007	♀	No
Heidi	2010	♀	No
Kiwi	2010	♂	Yes
Konrad	2010	♂	Yes
Mayday	2011	♀	No
Moneypenny	2010	♀	Yes
Muki	2011	♂	No
Muppet	2010	♂	Yes
Olympia	2010	♀	No
Pipin	2008	♂	Yes

Names of individuals are listed in the first column, with year of hatching and sex in the following columns; the last column (Touch screen experience) refers to whether individuals have participated in prior discrimination tasks on the touch screen.

All subjects were hand reared and obtained from European breeders in accordance with the CITES regulations. The daily diet consisted of basic food (Australian Parrot Loro Parque Mix supplemented with a selection of dried fruits) and fresh drinking water ad libitum and additional fresh fruit, soy yoghurt, eggs fried in red palm oil or cooked grains, noodles, vegetables in the mornings.

### Apparatus

The touch screen setup was an adapted mobile version of the operant conditioning system described by Steurer et al. [41]. The mobile version combined touch screen, CPU (based on a Schneider A4F minicomputer (http://www.mappit.de) with Mini-ITX main board (VIA EPIA1 M10000, with 1-GHz CPU, 2 × USB, 1 × LAN 10/100 Mbit, sound, and VGA on board), 512 MB DDR RAM, a 40-GB 2.5-in. hard disc) and automatic feeding system in one sealable aluminium cube (385 mm x 500 mm x 610 mm) with touch sensitive screen and reward tray (60 mm x 60 mm x 20 mm) located in the front and a flap on the back allowing access to a second screen, keyboard and mouse. A feeding wheel was attached behind the touch sensitive screen. Whenever a stimulus with positive contingency was touched the wheel would rotate so that a reservoir released a reward into the reward tray below the screen. The screen, a 15-inch XGA color TFT LCD Modul (Model G150XG01 by AU Optronics Corp., Taiwan; http://www.auo.com), provided a display area of 304 mm × 22 8mm (381 mm diagonal) and a resolution of 1024 × 768 px. A 15-inch IR “CarrollTouch” touchframe (Model D87587-001, 15 in., without filter) by Elo (Menlo Park, CA; http://www.elotouch.com) was attached to the screen in order to detect responses. The opening for delivering the reward was centrally located, 80 mm below the lower edge of the screen.

**Fig 1:**
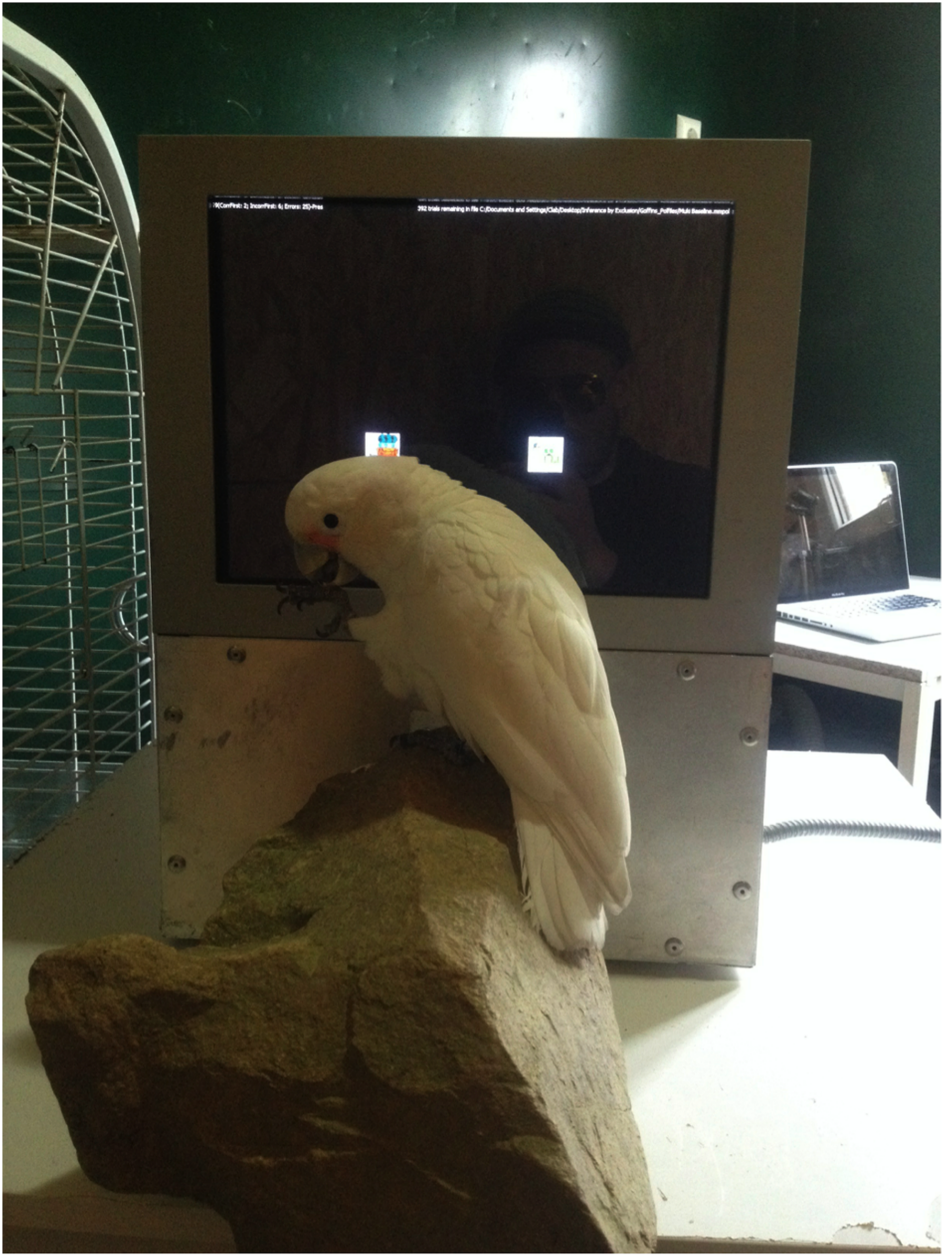
Experimental setup. Individual perching on the pedestal stone in front of the touch screen.

The touch screen apparatus was placed on a table (1 m x 1 m) with a stone placed in front of it. This stone (approx. 40 cm x 20 cm x 30 cm) served as a perch for the birds. It was approximately the same height as the reward tray, so the birds could easily access the stimuli and the food reward (Fig 1).

The program used for cognitive testing was CognitionLab (version 1.9; see [41] for a detailed description).

### Stimuli

**Fig 2:**
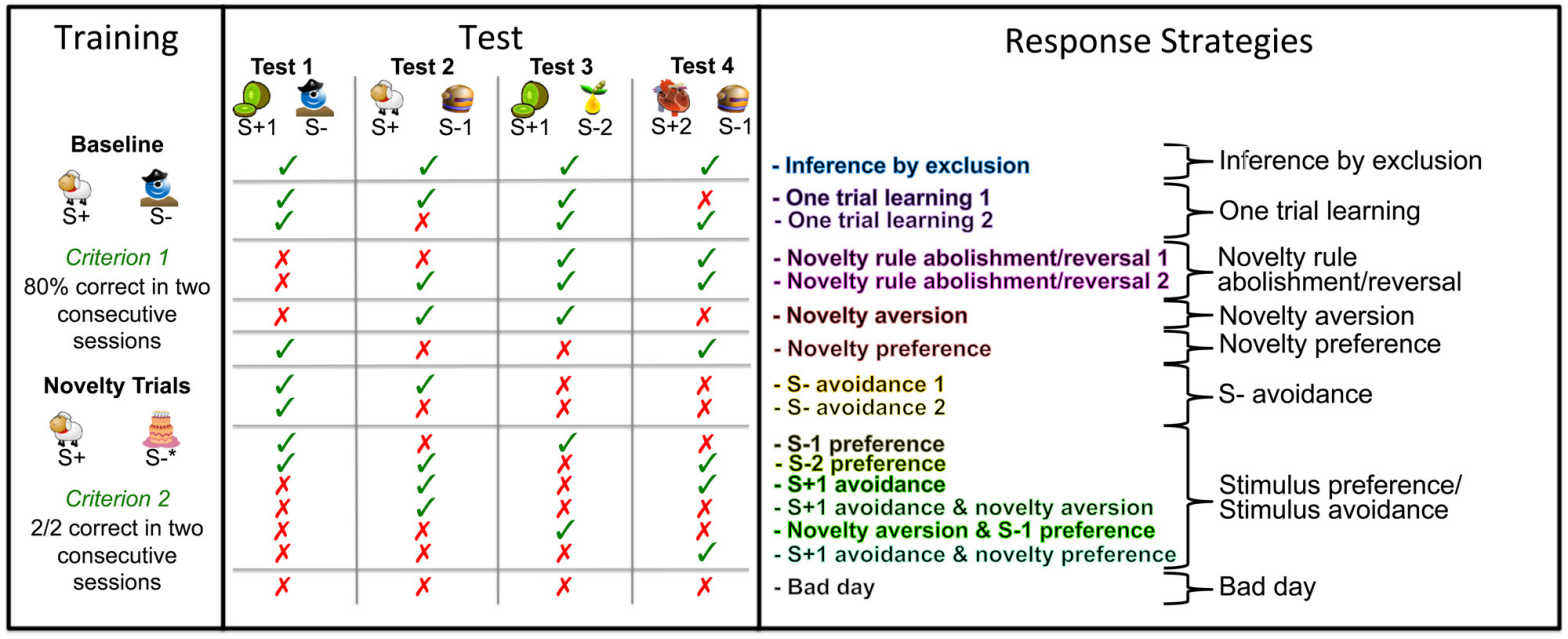
Experimental Overview. Schematic representation of one of each baseline trials, novelty trials and test trials with example stimuli, as well as theoretical response predictions (see main text) colour-coded to match response patterns in Fig 4; + indicates stimuli with positive reward contingency,—indicates unrewarded stimuli; ✓ correct, ✗ incorrect choices. On the right we list the most parsimonious mechanisms (see main text).

We downloaded a collection of license and restriction free clip arts from the Open Clip Art Library (http://www.openclipart.org/) as Scalable Vector Graphics (SVG). An arbitrarily chosen pool of 190 clip arts (see Fig 2 for an example) were resized to images on white background measuring 70 x 70 px, adapted for equal overall brightness and converted into Portable Network Graphic (png) files using Fiji (ImageJ 1.49e, http://imagej.nih.gov/ij; ImageJ 2.0.0-rc-9, http://developer.imagej.net/).

Two stimuli were presented simultaneously on the screen. They were positioned 484 px from the top and either 341 px (left) or 682 px (right) from the left side of the screen. In each trial, the program randomly placed the rewarded and unrewarded stimuli at either position. The background colour of the screen during the task was set to black (R = 0, G = 0, B = 0).

### Procedure

#### General Procedure

To separate individuals, the birds were individually called into the testing compartment by name. After the birds entered the compartment, a sliding door separating the aviary and testing compartment was pulled shut. Any additional bird that had entered the compartment had to perch on the experimenter's hand and was then released back into the group aviary. Except for the habitutation phase (see below) each session consisted of 20 trials and usually one session per individual was conducted per day, except for weekends when the experimenter was present all day and occasionally up to four daily sessions were provided (depending on subject motivation).

Correct choices were rewarded with small pieces of cashew nuts, automatically dispensed by the feeding system implemented in the apparatus. During each session, the experimenter (MO) was present outside the view of individuals but remained motionless, wore mirrored sunglasses and did not pay attention to the responses, so as not to cue the test subject. The experimenter’s presence during the task was kept consistent for all birds to minimize potential fear reactions towards the apparatus, since some individuals initially flew to the experimenter after first encountering the apparatus and would not perform when left alone in the testing compartment.

After each session the individuals had to step on the experimenter's hand and were then transferred back to the group. In the absence of the subjects the screen was wiped to remove stains and potential olfactory cues, and the apparatus was re-baited. The experiment was conducted four days a week between 5. March and 25. June 2014.

#### Habituation

Naive individuals were habituated to the touch screen and reward system by presenting a white square (70 x 70 px) centrally on the screen. Once individuals started to touch the square (with verbal and gestural encouragement from the experimenter (MO)) a reward (a twelfth to a sixteenth of a cashew nut) would be delivered into the reward tray. As soon as the stimulus was touched reliably in consecutive trials, the square was presented at random positions for 32 trials.

#### Training

For each individual, two stimuli (hereafter: the baseline pair) were randomly assigned a positive and a negative contingency, respectively. Responses directed at the positive stimulus (S+) resulted in the delivery of a reward, paired with a short sound (665 Hz, 200ms) and a time-out of one second during which the stimuli disappeared. Responses to the negative stimulus (S-) produced a shorter and lower pitched sound (405 Hz, 151ms) and led to a two second time-out during which the stimuli disappeared. Thereafter, the same trial was repeated as a correction trial until the individual touched the positive stimulus (S+).

Each session consisted of 20 trials in which two 'novelty trials' were semi- randomly interspersed (the first novelty trial randomly occurred in trials four to eight, the second randomly in trials thirteen to seventeen). In these novelty trials the S+ was presented together with a novel unrewarded stimulus (S-*), which was different for every novelty trial. Responses to the S-* had the same effect as responses to S- (low pitch sound, two second time out and correction trial). To ensure that the birds had learned the discrimination, the birds had to reach 80% correct first choices (15/18) in the baseline trials in two consecutive sessions (Criterion 1). Individuals were also required to completely inhibit responses to novel stimuli during all novelty trials (2/2) in two consecutive sessions (Criterion 2). The second criterion was introduced to ensure that responses to novel stimuli were not committed purely out of a neophilic tendency.

#### Testing

After reaching both criteria, the cockatoos were exposed to 25 test sessions, each in turn consisting of 20 trials. Four test trials were pseudo randomly distributed among 16 training trials (test trial 1 substituting trial 4 or 5, test trial 2 substituting trial 8 or 9, test trial 3 substituting trial 12 or 13 and test trial 4 substituting trial 16 or 17), but always occurred in the same sequence. For all test trials, stimuli with positive contingency were rewarded (as the S+), whereas responses in test trails to stimuli with negative contingency were immediately aborted (no sound) and followed by the next baseline trial.

In the first test trial the S+ was replaced with a novel positive stimulus (S+1), while the S- remained the same as in the baseline trials. In the second test trial a novel unrewarded stimulus (S-1) replaced the S- while the S+ remained the same, much like the novelty trials in training sessions. In the third test trial the novel positive stimulus of test one (S+1) was shown, now paired with a novel negative stimulus (S-2). In the fourth test trial the negative stimulus of test trial two (S-1) was presented together with a new positive stimulus (S+2; see Fig 2 for a schematic outline of a training and test session). For each test session a novel set of S+1, S-1, S+2 and S-2 was provided to avoid any learning effects other than one-trial learning. For each response pattern we tried to devise the most parsimonious explanation possible:

For answers to be classified as 'Inference by exclusion' individuals had to choose the novel positive stimulus (S+1) in the first test trial by inference or avoidance of the known S-, and continue to choose the S+1 in test trial three, by one trial learning or inference. Correct responses in test trials one and three therefore are indicative of inference by exclusion but alternative mechanisms are possible. However, correct responses in test trial two, either by choosing the stimulus with the greater associative strength, or possibly by inference, mean that individuals have gained no direct information about the contingency of the novel S-1 (as this stimulus was never encountered before and was not chosen). In such a case, individuals are faced with two stimuli (S-1 and S+2) with unknown contingencies in trial four. The only difference between these stimuli is that one (S-1) was previously presented with the S+ in test trial two. Individuals choosing the 'more' novel stimulus (S+2), counteracted what was rewarded for in the training, indicating an avoidance of the S-1 solely for contextual reasons, which can be considered an instance of reasoning by exclusion. Thus we employed a very strict criterion for a performance to be considered as representing 'Inference by exclusion': only if the correct stimuli (S+) were chosen in all four test trials.

We assumed that 'One trial learning' occurred whenever individuals did not directly infer the contingency of a stimulus without direct feedback (making a mistake solely in test trial four—'One trial learning 1'), or showed correct responses after one incorrect choice in test trial two ('One trial learning 2').

Other strategies may have been related to the relative novelty of the stimuli. Since the training included trials in which novel stimuli were not rewarded, it is possible that the subjects based their decisions on a general rule of novelty aversion: avoid the novel stimuli in the first and fourth test trial and choose the less novel stimulus in the third test trial. The reverse pattern would indicate instances of novelty preferences.

Furthermore, individuals may initially avoid the novel stimulus, but from then on choose the S+ ('Novelty rule abolishment/reversal 2'). Upon not receiving a reward in the first test trial, individuals may even reverse this aversion and choose the novel (but incorrect) stimulus in the second test trial, but respond 'correctly' in the last two test trials ('Novelty rule abolishment/reversal 1'). Therefore we labelled these two strategies 'Novelty rule abolishment/reversal'.

We currently have no explanation for the case when subjects choose the incorrect stimuli in all test trials (which occurred only once), hence we labelled this as a 'Bad day'. Other response patterns we attributed to combinations of stimulus preferences or stimulus aversions (see Fig 2 for an overview of response patterns and corresponding strategies).

### Data analysis

In order to assess performance on an individual level, we calculated the cumulative probability of each response pattern occurring by chance. Four test trials per session yield 16 possible different response patterns (see Fig 2). Therefore the cumulative probability for each pattern to occur by chance is p = 0.0625. This means that if individuals choose randomly, they should exhibit each pattern 1.5625 times over the course of the 25 test sessions. We employed a two-tailed binomial test to test whether the observed patterns were chosen more often than predicted by chance, which would imply preferences for certain strategies by producing consistently recurring patterns. Depending on how many response patterns a certain strategy predicts (see Fig 2), the probability for a strategy to occur by chance would increases by a factor of n (where n is the number of response patterns constituting a strategy). Strategies composed of only one response pattern (such as Inference by exclusion, Novelty aversion, Novelty preference and Bad days) therefore are statistically significant at p = 0.018 if the same pattern occurred in five of the 25 sessions. Strategies consisting of two patterns (such as One trial learning, Novelty rule abolishment/reversal, and S- avoidance) would need to be exhibited in seven out of 25 sessions to differ significantly from chance (p = 0.03). As Stimulus preferences/Stimulus avoidances account for six different patterns, this strategy requires to be displayed in 15 out of 25 sessions to reach significance (p = 0.023).

In order to investigate the effect of different factors on exclusion skills, incidents of Inference by exclusion were scored as successes, while all other strategies employed were considered failed attempts. We then applied a generalized linear mixed effects model with binomial error distribution and conditional log-log-link function to allow for asymmetry in the distributions. Including individuals as random factors this model allowed us to test for the influence of age (year of hatching), sex and prior touch screen experience by single term deletion from a full model and likelihood ratio testing. To investigate whether individuals had learned to infer by exclusion, rather than spontaneously applied their reasoning skills, we additionally included session as a fixed factor in our model. Furthermore, we included number of sessions required to reach Criterion 1 as well as Criterion 2 as a factor, to examine whether the time required to learn to inhibit responses to novel stimuli in the training influenced exclusion performance.

To investigate the effect of different response patterns on a group level we employed a generalised linear mixed model with assumed Poisson distribution. As in the binomial model we included sex, age, sessions to reach Criterion 1 in training (learning the baseline discrimination) and sessions to refrain from pecking on novel stimuli during training (Criterion 2). We performed Wilcoxon signed-rank tests for pairwise comparisons of all patterns with the pattern suggestive of Inference by exclusion.

Binomial exact tests were conducted in the statistical package R [49], models were fitted employing 'lme4' [50] and post-hoc adjustment for the Intercept using chance probability of 0.0625 was achieved by using the 'esticon' function of the package 'doBy' [51]. Visual representation of the data were created with the package 'ggplot 2' [52].

## Results

**Fig 3:**
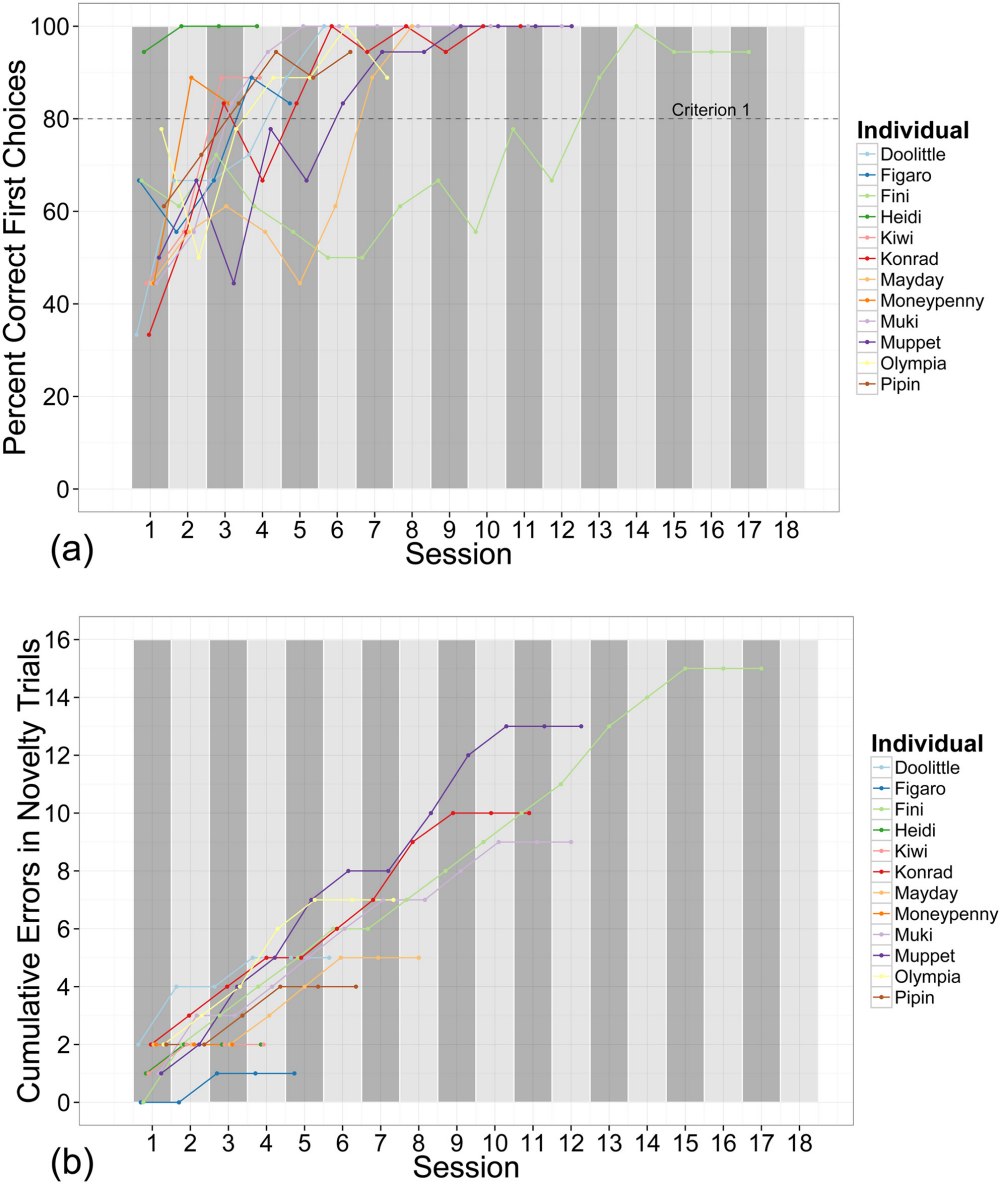
Performance in the training phase. a) Learning curves for all individuals over all training sessions, with the dotted line indicating the learning criterion of 80 per cent correct first choices (Criterion 1); b) Cumulative errors of novelty trials; a steep incline indicates novelty responses in both novelty trials, moderate incline indicates response to one novel stimulus per session and a straight horizontal line indicates no responses towards novel stimuli (Criterion 2); longer lines indicate individuals required more sessions to inhibit responses to novel stimuli.

Seven individuals learned the discrimination of the baseline stimuli (Criterion 1) before ceasing to respond to the novel stimuli (Criterion 2). Two individuals managed to inhibit their responses to novelty before reliably discriminating the baseline stimuli, and three subjects reached both criteria simultaneously. Overall, subjects required on average 7.92 sessions (+/- 1.22 SE) to complete the training phase. Individuals required on average 5.67 sessions (+/- 0.90 SE) to learn the discrimination of the baseline stimuli, whereas it took them on average 7.25 sessions (+/- 1.35 SE) to refrain from selecting the novel stimuli. All individuals chose a novel stimulus at least once (*M* = 6.25, *+/-* 1.33 SE; see Fig 3).

**Fig 4:**
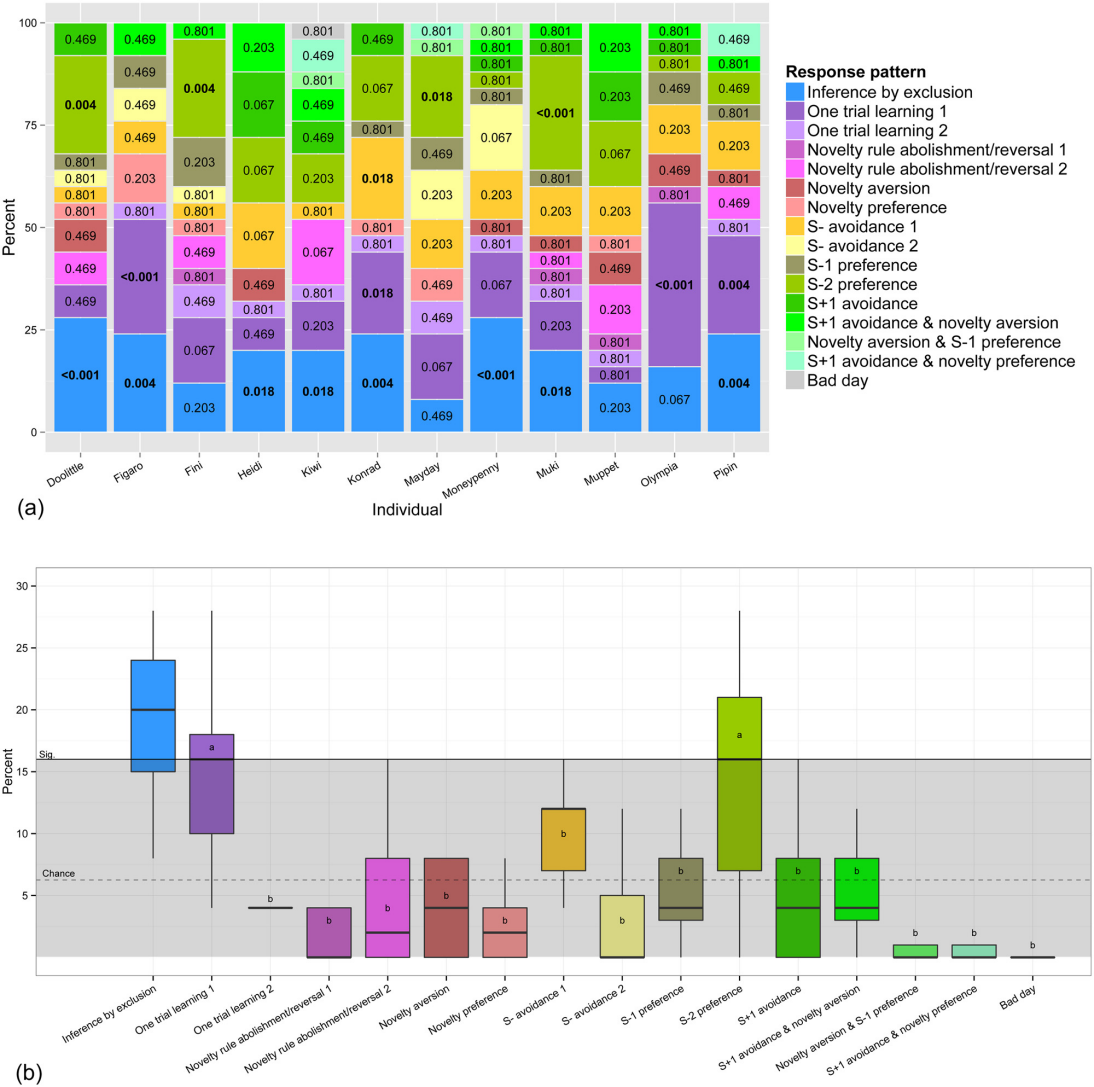
Percentages response pattern frequencies. a) Detailed patterns exhibited by each individual in all 25 test sessions; values in the bar graphs correspond to the probability that the number of sessions with certain response patterns occurred by chance; significant values are printed bold; b) Frequency of patterns exhibited at group level; horizontal lines indicate median values, boxes span the first to third quartiles and whiskers represent 95% confidence intervals. The dashed horizontal line represents chance levels; the grey area below the line denotes no significant divergence from chance. The letters in the boxplots refer to a Wilcoxon signed-rank test comparing patterns to 'Inference by exclusion'—a: no significance, b: significant, p< 0.05.

At the individual level, eight individuals exhibited the Inference by exclusion response pattern significantly more often than predicted by chance (see Fig 4a). The only other patterns that also occurred above chance level were One-trial learning, displayed by Figaro, Konrad Olympia and Pipin, as well as a preference for the second novel negative stimulus (S-2), which was shown by four individuals (Doolittle, Fini, Mayday and Muki). One individual (Konrad) seemed to have established an additional rule to avoid the unrewarded baseline stimulus (S-). It is important to note that these patterns are not mutually exclusive, as Figaro and Pipin also relied on inference by exclusion and one trial learning significantly, while Doolittle and Muki showed inference by exclusion and a preference for the S-2 significantly above chance. Konrad even simultaneously exhibited Inference by exclusion, One trial learning 1 and Avoidance of the unrewarded baseline stimulus at statistically significant levels.

Binomial modelling, with regard to sessions showing Inference by exclusion, revealed a significant intercept (GLMM: *b* = -1.52, SE = 0.13, *X* ^*2*^(1) = 83.12, *p* < 0.001), but no significant effects of sex (*X* ^*2*^(1) = 0.003, *p* = 0.956), age (*X* ^*2*^(3) = 1.56, *p* = 0.670) or prior experience on the touch screen (*X* ^*2*^(1) = 2.77, *p* = 0.096) on the occurrence of this response pattern. We found no influence of session (*X* ^*2*^(24) = 25.43, *p* = 0.383) discrimination-learning performance in the training (*X* ^*2*^(1) = 1.76, *p* = 0.185), or inhibition of novelty responses during training (*X* ^*2*^(1) = 0.03, *p* = 0.860).

Generalised linear mixed models investigating how often each pattern occurred revealed only a significant effect of response patterns (*X* ^*2*^(15) = 224.97, *p* < 0.001). Post hoc tests revealed that Inference by exclusion occurred significantly more often than other response patterns, except for 'One trial learning 1' and preferences for the S-2 (see Fig 4b). Furthermore, when comparing frequencies of patterns for 'Inference by exclusion' and 'One-trial learning 1' only for those individuals that exhibited exclusion performance above chance, significantly more 'Inference by exclusion' (*M* = 5.88, *+/-* 0.24 SE) than 'One trial learning 1' (*M* = 4.00, *+/-* 0.53 SE) was employed on a group level (Wilcoxon signed-rank test: *p* = 0.048; *r* = -0.51).

When grouping the individual patterns to hypothesized response strategies (see Fig 2), combinations of stimulus avoidances and preferences, consisting of six possible response patterns, were exhibited on average in 32% of the test sessions (*SE* = 2.83). Furthermore, subjects seemed to rely on one-trial learning (*M* = 21.00%, *SE* = 2.79), inference by exclusion (*M* = 19.67%, *SE* = 1.87) and avoidance of the S- (*M* = 14.33%, *SE* = 2.00).

## Discussion

Two thirds of the subjects exhibited inference by exclusion by showing the corresponding response pattern in significantly more trials than predicted by chance. Since novel test stimuli were used in each test-session, experience cannot account for these results. However, an immediate association formed with the S+1 in the first test trial (by avoiding the known S-) could have underlain choices in test trial three, through one-trial learning. Such one trial learning, or rather 'One trial avoidance', cannot, however, account for correct responses in test trials two and four. Crucially, if individuals chose correctly in the second test trial, which was a prerequisite for being considered an instance of inference by exclusion, they received no direct feedback about the unrewarded (but not chosen) stimulus. Thus the contingency of the S-1 remained unknown to the individual, unless avoidance of this stimulus occured for contextual reasons (being presented in combination with a rewarded stimulus), which would be considered an exclusion performance.

An alternative explanation for patterns of 'Inference by exclusion' might consider correct choices in trials one through three, based on associative strength and avoidance of the S-, and a random choice in the fourth test trial. If so, there should be similar frequencies of patterns for 'Inference by exclusion' and 'One trial learning 1'. While this might have been the case for three individuals (Figaro, Konrad and Pipin; see Fig 4a), it is unlikely to apply to all individuals who exhibited successful exclusion performance. For those individuals who showed well above chance 'Inference by exclusion' patterns, we found a significantly higher frequency of 'Inference by exclusion' patterns than of 'One trial learning 1' at a group level. Therefore we conclude that at least those five individuals (Doolittle, Heidi, Kiwi, Moneypenny and Muki) chose the rewarded stimulus (S+2) of the fourth test trial by inferring that S-1 was unrewarded. In this crucial test, no other cues were available to evaluate the contingency of both stimuli.

These inferential responses did apparently not emerge as a consequence of incremental learning because session, as a factor, had no effect on the response patterns. Since the performance in the training (sessions required to reach Criterion 1 and Criterion 2) also did not affect inferential patterns during testing, we conclude that prior experience in the training, as well as experience during testing itself did not influence this ability. Thus, we suggest that at least five subjects were able to spontaneously solve the task in an inferential manner.

However, this does not mean that Goffin cockatoos respond entirely logically in categorization tasks like the present one. The individuals also exhibited other strategies with novel stimuli: One-trial learning, stimulus preferences and avoidances, as well as the avoidance of the S-, represent further strategies. From a different perspective, these strategies—one-trial learning and avoiding the unrewarded stimulus and stimulus preferences—can also be considered efficient. Learning to categorize novel items on the basis of a single encounter in this task requires high levels of cognitive plasticity, given that during training individuals were required to inhibit responses towards novel stimuli in order to proceed to testing. Preferences might emerge when test stimuli have a similar colour or shape as the S+. Ecologically, it would be adaptive to seek out items, which resemble other items that were previously rewarded and to avoid items similar to ones that were not. However, only one individual (Konrad) showed a significant avoidance pattern for the unrewarded baseline stimulus.

In humans, the prefrontal cortex and in particular the inferior parietal lobule play a major role in inference by exclusion tasks [6]. Since these brain areas have also been associated to language learning and tool use in primates [53], inferential reasoning has been suggested to be a uniquely human trait [5,6]. The present study as well as Aust et al. [7] provide cumulative evidence against this anthropocentric view. A very recent study by Nawroth and colleagues [26] further supports the argument of exclusion performance not being uniquely human by showing that goats (but not sheep) are able to successfully choose the position of a food reward by exclusion. The authors discussed the found species difference with respect to different feeding ecology, especially different foraging strategies, similarly as Schloegl et al. [30,31,36] did for different corvids and kea and Mikolasch et al. [33] suggested for carrion crows.

Goffin cockatoos are highly inquisitive and playful in captivity [43]. This may explain why most individuals kept responding towards the novel stimuli even though they had already reached the criterion in the initial discrimination task and had formed a positive association with the S+. Our data therefore challenge the hypothesis proposed by Schloegl et al. [30,36] that exclusion performance may be missing in highly explorative birds and may rather have evolved as an adaptive consequence of intraspecific competition during foraging in some species such as corvids. While this might be true for some corvids, a recent study showed that Eurasian jays (*Garrulus glandarius)*, a corvid species which habitually stores food items, failed this task [34], contradicting Schloegl et al.'s hypothesis. Mikolasch et al. [33] showed that contradicting social cues can overshadow exclusion performance in crows, which potentially also may have led to the Eurasian jay's failure [34]. However, considering the overall body of evidence, food-storing behavior cannot be the sole source of exclusion skills.

Given that the ability to infer by exclusion has been found in distantly related species with different ecological backgrounds, including humans [6,7,12], nonhuman primates [4,8–11,13–20], dogs [20–22], goats [26], ravens [30], carrion crows [33], New Caledonian crows [27], African grey parrots [28,29,32] and now Goffin cockatoos, we suggest that its repeated emergence is the result of convergent evolution. It is yet unclear why the ability seems to be missing in pigeons [7], sheep [26] jackdaws [31], and Eurasian jays [34], but some of the latter results may be influenced by methodological issues in past task setups.

In this study we showed that Goffin cockatoos are able to perform inferences not only in the technical domain [47], but also exhibit exclusion skills in very abstract tasks. Whether the Goffins' competence in physical cognition gave rise to the ability to infer by exclusion and also allowed them to apply this skill in different contexts, or whether this skill evolved due to other selective pressures and drove their advances in the physical domain remains speculative. The latter explanation would apply according to Pepperberg et al. [29], who suggested that exclusion might be a fundamental skill that developed in different species due to similar selection pressures and which ultimately makes the emergence of even more sophisticated cognitive skills possible.

However, precisely which environmental or social factors drove the emergence of the trait is difficult to say. The selection pressures that shaped the evolution of this ability in different species currently remain speculative and will have to be the subject of further comparative studies. We believe the testing paradigm introduced here has great potential to investigate inferential reasoning by allowing an evaluation in comparison to both, other species and alternative response strategies.

## Supporting Information

S1 Table Complete dataset. (XLSX)
